# Free Will, Religious Conflict, and the Social Contract

**DOI:** 10.1007/s10790-023-09931-y

**Published:** 2023-02-14

**Authors:** Luke Christopher Armstrong

**Affiliations:** University of Glasgow, Glasgow, UK

## Introduction

Saul Smilanksy has written that John Rawls’ theory of justice is ‘infused with the assumption of free will and moral responsibility’.^1^ If this remains true of the conception of justice political liberals hold, political liberalism is failing in its task to remain neutral between competing metaphysical doctrines.^2^ Hard determinists and incompatibilists may argue that their doctrines conflict with this conception of justice. Nevertheless, metaphysical disagreements between people on the subject of free will tend not to be the source of major conflicts in modern societies. The purpose of abstaining from metaphysical commitments is to avoid exacerbating such conflicts. Should we worry, then, if a political doctrine contains an implicit metaphysical assumption regarding free will? Would these metaphysical assumptions make the attainment of a social contract impossible?

While metaphysical perspectives on free will tend not to be a direct source of conflict, there are nevertheless such perspectives within many religious doctrines, which are sources of conflict. Thus, if the public reason upholding a constitutional consensus implicitly accepts the truth of free will, the religious doctrine which rejects free will may seem to be disadvantaged. If this is true, then, perhaps we should ensure public reasons do not contain such metaphysical assumptions.

However, there seems to be a fundamental tension between the position of hard determinists who deny moral responsibility and justice as fairness. These hard determinists may deny the possibility of some of what Rawlsians demand of the citizen, such as formulating her own conception of the good and bearing the consequences of this conception. This reveals the difficulty of keeping political liberalism ‘political’. The political judgements we make may often contain metaphysical assumptions that are in tension with the beliefs held by many other citizens. In this paper, I argue for two central contentions. First, there are often implicit metaphysical assumptions within political principles; these assumptions may jar with what some reasonable citizens believe to be metaphysically true. Second, that this should not be considered problematic. What matters is a social contract based on fairness – fair *in terms of deliberation*, not necessarily distribution – with citizens being committed to upholding this ideal, regardless of other moral and metaphysical conflicts. Nevertheless, these conflicts should not be ignored. What citizens hold to be metaphysically true can alter the way in which political concepts are formulated; providing those who reasonably disagree are respected, these alterations should be embraced rather than avoided.

In Section 2, I briefly explain why political liberals claim a social contract should not be devised on metaphysical grounds. Then, in Section 3, I explain the three dominant metaphysical perspectives on free will – libertarianism, compatibilism, and hard determinism – before turning to the role these perspectives play in religious doctrines in Section 4. Through doing so, I attempt to show that, though debate on free will is not a source of direct conflict, this debate nevertheless plays a role within religious conflict. In Section 5, I explore what this means for the social contract and its justification. Whether or not Smilansky is correct that justice as fairness presupposes the truth of free will, I argue there is a tension between justice as fairness and hard determinism. I explore four possible resolutions for this tension, before identifying a more promising solution in Section 6. This is then further explained in Section 7, where I argue that this leads to a challenge to political liberalism’s position on the relationship between metaphysics and politics. Those who disagree with the metaphysical assumption contained within an accepted political principle need not be dismissed as unreasonable and excluded from the scope of political justification. Instead, it is a matter of how we convince those who disagree or learn to live with disagreement. As long as people are committed to the importance of fairness in deliberation, a social contract based on this principle is realisable. The metaphysical implications of a political principle do not necessarily challenge the realisation of a social contract based on this principle; instead, they grant further depth to the support for this principle.

## Metaphysics and Politics

I begin by briefly explaining the problem of considering metaphysics within politics. Political liberals take the view that using metaphysical reasoning to support political principles will lead to societal divisions. As people within modern societies hold a variety of philosophical and religious beliefs, appealing to the truth of any one belief in support of a political principle will mean those with alternative beliefs refuse to support the political principle. If we hope to attain a consensus regarding the political principles we choose to uphold the constitution, and the social contract more generally, the reasons we use to support these principles should be free of metaphysics. In this section, I explain the basic problem of using metaphysical reasoning to support a political principle.

In ‘Justice as Fairness: Political not Metaphysical’,^3^ Rawls sets out his view that his conception of justice – justice as fairness – is not dependent on a metaphysical truth. Rawls states that justice as fairness does not make ‘claims to universal truth, or claims about the essential nature and identity of persons’.^4^ These are the sorts of metaphysical truths we should avoid when making the case for a political principle, according to Rawls. A state that made claims about ultimate truths and lacked toleration of other viewpoints would be an autocratic one. Thus, Rawls argues that the reasons used to support a liberal democratic order should not appeal to such truths.^5^ This is, then, a practical task, not a metaphysical one. In attempting to reach a consensus, we seek to use only reasons to which we might expect others to agree.

This line of thought encompasses what Rawls and others call ‘public reason’. Public reason places limits on the kinds of arguments which are appropriate to make in the course of political deliberation. When debating constitutional essentials – basic rights and liberties, the structure of government, for example – we should not appeal to universal truths, but to reasons embedded within the public political culture we can expect other citizens to accept.^6^ Such a reason is a public reason. The opposite of a public reason, a non-public reason, is not limited in the same way.^7^ To use Rawls’ examples, when the members of a church discuss an aspect of a theological doctrine, educational policy is discussed within a university, or scientists discuss the harm to the public resulting from a nuclear accident, they can appeal to non-public reasons. The process of reasoning used to determine the conclusion need not be subject to the constraints of public reason as there is no question of political force or coercion involved. Those who disagree with the conclusion are free to leave the association. Citizens subject to the use of political power need to be able to agree with the reasons used to justify this power.

This is, then, why metaphysical truths should be avoided throughout the course of political deliberation. For political liberals such as Rawls, Jonathan Quong, and Samuel Freeman,^8^ appealing to the truth of a particular metaphysical doctrine in support of a political principle would be to step beyond the limits of public reason. Hence, the political liberal would not appeal to the truth of a particular metaphysical perspective on free will to support a political principle. As it is unlikely every citizen in a modern society would share this same perspective, to argue for a political principle on these grounds would be to risk deepening divisions in society and breaking the social contract.

## Why We Might Reject Free Will

While some philosophers endorse compatibilism or libertarianism, arguing that we do have free will, there are those who reject free will. In what follows, I explain their reasoning before offering definitions of some of the terminology within the metaphysics of free will.

Before explaining hard determinism, it is necessary to understand what is meant by determinism. The thesis of determinism states that from one moment in time to another there is only one possible eventuality.^9^ Someone accepting determinism endorses the view that in the universe, at any given moment in time and in any situation, one event will necessarily lead to another event, and this could never have been otherwise. Everything is subject to the laws of physical causation; all of the events that occur throughout time and space are necessitated by the events that preceded them. This is true at all levels of matter, from the sub-atomic level upwards.^10^

The first philosopher to notice the antinomy between determinism and free will was Epicurus,^11^ who modified his account of determinism to allow for human free will. Others think that determinism negates the possibility of free will completely. Hard determinism is the view that determinism is true, and as a result, we do not have free will. The laws of nature, and of physical causation, determine how any event unfolds. Human actions are no different. We had no control over past events, nor do we have any control over the laws of nature.^12^ Therefore, we have no choice in regard to the actions we make. The hard determinist argues we could never have acted other than we did. As our inner mental states are the result of laws over which we have no control, we cannot be the original creators of our own ends, meaning we cannot freely choose our actions. We neither have control over the constitution of our inner motivations nor what we choose to do because of these motivations. For hard determinists, this means we do not have free will. Some contemporary philosophers accept that determinism may be false, but argue this does not build a better case for free will. Calling himself a ‘hard incompatibilist’, Derk Pereboom argues that indeterminism is equally hostile to free will.^13^ If all events are subject to chance outside of agent causation, we would not be acting of our free will but as a result of chance circumstances outside of our control.

These are some reasons why we might deny that the will is free. Not all philosophers are convinced. Libertarians argue that there is just enough indeterminism within the universe to allow us to have free will. Robert Kane has set out a theory of libertarianism which has become dominant within contemporary libertarian metaphysics.^14^ Other philosophers endorse compatibilism, the position that free will and determinism are not necessarily at odds. Thomas Hobbes and David Hume developed some of the first accounts of the compatibilist position,^15^ while in contemporary times, Harry Frankfurt has established a defence of compatibilism which has become dominant,^16^ and John Martin Fischer argues for a position he calls ‘semi-compatibilism’.^17^ These philosophers offer several accounts of how free will and determinism are compatible. For Hume, determinism is a prerequisite for free will; it is because determinism is true that there is a necessary connection between the will and the act. For Frankfurt, it is not the availability of alternatives that allows us to have free will, but our ability to reflect on what he calls our ‘first-order desires’. It is because humans do not act on impulse but can deliberate that they are free; humans can do this regardless of whether determinism is true. Fischer finds some of the hard determinist position convincing, but follows Frankfurt in arguing that compatibilism salvages moral responsibility.

In sum, libertarians and compatibilists agree we have free will; however, they disagree on the precise ways in which we have free will; while the compatibilist views free will as independent of the issue of determinism, libertarians argue that in order for free will to be true, determinism must be false. Hard determinists agree with the premise of libertarianism – determinism negates free will – though the hard determinist inverts the argument, and states that because determinism is true, we cannot have free will.

## Religion and Free Will

While it seems unlikely a modern society could be deeply divided by the perspectives people took on the question of free will – the doctrinal conflicts in modern societies tend not to be premised directly on the metaphysics of free will – there are positions on free will within religious doctrines. In this section, I assess positions on free will within theology, establishing how these positions relate to contemporary debate on free will. This is to show how premising a political principle on metaphysical assumptions regarding free will could lead to tension between the political principle and certain religious doctrines.

Christian theologians such as Saint Augustine recognised a similar problem in Christianity to that of the tension between free will and determinism; how to reconcile free will and moral responsibility with divine omniscience and predestination.^18^ There are a number of interpretations of Augustine’s solution to this problem. Only beings before the fall from grace, for Augustine, possessed the capacity for unhindered free choice, Adam and Satan both freely choosing to sin.^19^ Due to Adam’s sin, we are all born in sin. It is by God’s grace that we are able to resist evil and live the good life. Freedom consists only in the freedom to sin; when we act in accordance with the good, we do so due to divine intervention. It is, then, through God that we are able to act from the good. Once God has intervened, however, theologians dispute whether Augustine believed we have free choice to do as we wish.^20^ Nevertheless, for the metaphysical libertarian, Augustine grants us little room for free will, as our ability to do otherwise is dependent on God’s will. There are two ways of interpreting this. On the reading that argues Augustine believed we could do otherwise once God has intervened, Augustine can be characterised as offering a limited libertarian perspective on moral responsibility, though on Rist’s reading, Augustine is a moral determinist: when God has intervened, we can do no other. Augustine’s stance on free will, however, is closer to compatibilism. As we have a will, the will must be free, whether we act from good or evil.^21^ The notion of an unfree will would be a contradiction in terms for Augustine. That we have a will, and it is in our power to perform the object of our will, means that the will is free.

Thomas Aquinas, on the other hand, is an incompatibilist, according to Robert Kane, influenced here by Eleonore Stump.^22^ The will, for Aquinas, is the primary mover, according to Stump.^23^ Though the will receives advice from the intellect, the intellect, while it can influence the will, does not perform the executive role during decision-making. The will, receiving information on the external environment, and advice as to the best course of action given the conditions, is responsible for determining its own constitution. Though the intellect is influential in this process, the will is also in control of the intellect. The will wills toward certain objects and not others; it can also will itself. In this sense, the will, acting as the primary mover of all other aspects of the human body and psyche, is free. Contra Hume, Aquinas holds that the will can be free even when the act is restricted; freedom of action and freedom of the will are distinct for Aquinas. Even under restrictive conditions, the will still wills itself, and is therefore free. Stump characterises Aquinas as an incompatibilist, as the truth of determinism would negate free will in the Thomist view, on which the will is not determined by prior happenings but determines itself, even under the most restrictive circumstances.^24^

Against Aquinas, Martin Luther held that the will was not free.^25^ Luther’s view is closer to Augustine’s, though while Augustine still regarded the will as necessarily being free, Luther considers predestination and free will incompatible. For Luther, salvation came through Christ, not through the individual’s capacity to choose salvation for herself. Luther writes that the human will exists between the control of God and Satan, as a beast with a rider. ‘If God sit thereon, it wills and goes where God will’, Luther writes, but ‘if Satan sit thereon, it wills and goes as Satan will. Nor is it in the power of its own will to choose, to which rider it will run, nor which it will seek; but the riders themselves contend, which shall have and hold it’.^26^ Thus, Luther’s view is closer to the hard determinist position.

Perspectives on free will are similarly divided in other religions. In Judaism, Moshe Sokol writes that, traditionally, most have considered the Torah scholar Maimonides a libertarian, due to his insistence in several places that ‘human choice is undetermined’.^27^ However, Sokol notes that others see Maimonides as arguing that human choice is part of the natural order, an order determined by God. The solution Sokol proposes is influenced by Frankfurt’s compatibilism. Thus, we can find libertarian, compatibilist, and hard determinist readings of Judaism. William Watt writes that it has been commonly believed that Christianity allows for free will while Islam insists on predestination, but that this view underplays the complexity of the issue within each religion.^28^ As there are stark differences between the Augustinian, Thomist, and Lutheran accounts of free will, there are similar differences between different schools of Islam on the matter of free will. Watt demonstrates that while certain passages in the Qur’an explicitly condemn fatalism, some Islamic scholars encourage complete faith in predestination, rejecting human intervention in practices such as medicine and relying instead on the divine will.^29^ Other scholars of Islam, such as Radwan A. Masmoudi,^30^ stress the importance of free choice within Islam. Masmoudi posits that, according to the Qur’an, religion cannot be forced upon a person; a person must come to believe through their own free choice. This is not to say that liberal Islam is necessarily committed to a particular position on the metaphysics of free will, but that the interpretation of determinism that leads to fatalism, and abandoning the importance of free choice, is rejected by liberal Islam.

In Eastern religions – such as Buddhism, Sikhism, Hinduism, Jainism – the concept of karma holds implications for how we think about free will, as Robert Kane notes – ‘good deeds produce good Karma and lead to progress toward liberation; bad deeds do the opposite’.^31^ This leads us to the question of whether we have any choice in regard to committing good or bad deeds. Though karma relates to causation – it accrues across the course of multiple lives, meaning our current spiritual status is the effect of our acts in past lives – Kane notes that predestination and fatalism are generally rejected in Eastern religion; one Jainist text claims that ‘choice is everything’.^32^ If Kane is correct in his reading of these Eastern religions, the stance on free will they hold seems closest to the libertarian position. Though the causes that have led to the present moment are recognised, they must not completely determine the present moment.^33^ There must be some space left for the individual to make choices that are not only the result of past chains of causation.

Paul Hacker offers a similar reading within his assessment of karma’s role in Hinduism.^34^ Karma is immaterial and transcendental, according to Hacker. It offers us a ‘model of behavior’. When we act, karma is realised, becoming adharma; it also remains long after the act, following us throughout our existence. None of this is predetermined, according to Hacker: ‘there is always a realm of freewill in which new *karman*, new *dharma* or *adharma*, can be accumulated’.^35^ Hacker’s reading of Hinduism’s stance on free will is libertarian, much like Kane’s. Through our acts in past lives, we have determined our present state, but room remains for us to choose otherwise in this present state. By doing so, we create new karma.

Though this is far from comprehensive, it provides an overview of the diversity of perspectives on free will within religion. While there are libertarian, compatibilist, and hard determinist readings of Christianity, Judaism, and Islam, with Lutheranism and certain readings of Judaism and Islam leaning more towards hard determinism, Eastern religion tends towards libertarianism.

## Justice and Free Will

While Smilansky has argued that Rawls’ conception of justice is dependent on the truth of free will, my aim here is not to establish whether this is the case, but to question whether it would be problematic if it were the case. I begin this section by examining Smilansky’s argument in order to establish the case for why there may be a tension between the metaphysical implications of justice as fairness and hard determinism. The Rawlsian political liberal who aims to devise non-metaphysical political principles should find this problematic.

It is worth quoting Smilansky in full to convey his argument for why justice as fairness presupposes the truth of free will:

For, once Rawls sets up his order in a seemingly free will-free way (except for the merely negative hard determinist rejection of "pre-institutional desert"), his book is thoroughly infused with the assumption of free will and moral responsibility. People are expected to fulfill their obligations, to take responsibility for their actions, and indeed to try to form their expectations, in a way that makes sense only if non-hard determinist positions on the free will problem are assumed. Rawls makes the strategic decision to throw "metaphysics" out of the door, but then quietly lets it in through the window, where it rightly (and perhaps inevitably) becomes dominant.^36^

Thus, though Rawls begins his theory partially as a hard determinist, he ends by accepting the truth of free will, according to Smilansky. On Smilansky’s argument, if we are to hold duties, be responsible for our actions, and form our expectations of what our share of primary goods ought to be, we must have free will. If hard determinism was true and we failed in our duties, committed irresponsible actions, or formed unrealistic expectations, we could not have done otherwise. The ground on which Rawls expects us to act as morally responsible agents would be lost. One does not have to accept Smilansky’s argument in its totality to see that there is a tension between the views of the hard determinist and the Rawlsian.

Similarly, Richard Arneson states that Rawls:

wants to deny that we should set up institutions with the aim of rewarding the deserving, but neither does he wish to deny a role to individual agency and individual responsibility within his theory of justice. After all, the distinction between deep and shallow inequalities rests on the idea that individuals sometimes make voluntary choices for which they are responsible, such that it is morally appropriate that they bear the consequences for their lives that result from these choices.^37^

The hard determinist who rejects the truth of free will may reject the notion of voluntary choice: a person may have made choices, but as there was nothing voluntary about these choices – she could never have done otherwise, so there is no sense in which she ‘volunteered’ to make these choices – she should not be made to bear the consequences, whether good or bad. Such a hard determinist may, then, agree with the premise from which Rawls sets out – the rejection of distribution according to desert – but disagree with the conclusions Rawls reaches about individual responsibility. If laws were then premised on these conclusions, the hard determinist may feel disadvantaged. The hard determinist may feel that people should be protected from the consequences of bad choices, rather than bear individual responsibility. Leaving individuals to determine their own plans of life or to face the outcome of gambles which they have made could be considered unethical by the hard determinist, as the hard determinist thinks the individual has no choice over her plans or the gambles she takes. Thus, hard determinists may desire more interventions into everyday life.

G.A. Cohen notes that, in private conversation between them, Rawls expressed a sceptical attitude towards compatibilism.^38^ If everything was causally determined, Rawls doubted whether the types of moral judgements we often make would continue to hold. Cohen does not say, however, whether this led Rawls towards libertarianism or hard determinism. If Smilansky and Arneson are correct in their readings of Rawls’ theory of justice, Rawls slips between the two positions, beginning a hard determinist, but ending a libertarian.

Whether or not we accept these arguments, would it cause us trouble if they were true? At first sight, it seems that it would. If Smilansky is correct, justice as fairness is not entirely metaphysically neutral, as it forces us to accept the truth of free will. Through public reason, we could not expect those who rejected the truth of free will to accept justice as fairness.^39^ Hard determinists could not accept a doctrine which committed them to this truth. While we might imagine that metaphysicians who endorse hard determinism would be peeved by this, it seems unlikely this would undo the social contract. Nevertheless, Lutherans or those who endorse fatalistic interpretations of Islam may object to a public reason containing a tacit acceptance of free will. They might argue that this public reason cohered more with the doctrines of Thomism or Eastern religions such as Hinduism or Buddhism, which accept the truth of free will. Such a public reason may exacerbate existing religious divides.

It seems, then, that this is a problem. If we hope to achieve a non-metaphysical political doctrine which people can accept regardless of their other religious and philosophical convictions, this doctrine cannot contain a position on the truth or falsity of free will. How should this problem be resolved? There are a number of options available to us. First, we could follow Rawlsians in arguing that justice as fairness is not metaphysical, and thus does not hold implications for how we think about free will. However, if the metaphysical implications are admitted, there are a number of other options available. Second, those who endorse hard determinism could be cast as unreasonable. If a doctrine rejects moral responsibility due to the implications of hard determinism, the person who endorses this doctrine is being unreasonable. As Quong posits, the unreasonable person – the person whose beliefs and way of life are incompatible with life in a liberal society – is beyond the scope of political justification.^40^ There is, then, no need to justify a political doctrine to the unreasonable person. Third, a distinction could be drawn between metaphysical determinism and theological determinism. Fourth, a thinner view of agency could be proposed, eschewing any notion of responsibility. Finally, other means for justifying a political doctrine could be sought. This is, I argue, the most appropriate solution, and is further explained in the following section.

The first four options are, I think, unsatisfactory. First, the traditional response of Rawlsians is to argue that in their account of responsibility, no position on the truth or falsity of free will is assumed. Samuel Freeman argues none of the ways in which Rawls argues a person is free entail a commitment to free will:

Persons are responsible agents so long as their moral powers are developed to the requisite minimum degree. This is the ordinary idea of responsibility that is employed in legal contexts and in everyday moral contexts. The question of responsibility is a factual one depending upon the capacity for a person to act rationally in his or her own interests, and understand, apply, and conform to moral and legal rules.^41^

Thus, at least according to Freeman, political freedom is in no way related to metaphysical freedom. Whatever our stance on metaphysical freedom, we should be able to agree to this political conception of freedom. Despite this, Freeman then states:

But as a result he must take a position by default on the moral question whether moral responsibility is compatible with determinism – clearly he thinks it is, since he regards the metaphysical question as irrelevant to moral responsibility.^42^

Freeman concedes, then, that the political conclusions we reach in regard to freedom and responsibility can conflict with certain metaphysical positions. Whether or not Smilanksy is correct that justice as fairness is committed to the truth of free will, a hard determinist may object to any doctrine that ties us to moral responsibility. The hard determinist could argue that moral responsibility is impossible due to the falsity of free will, and that we cannot, therefore, be deemed responsible for our duties, acts, or expectations. As Derk Pereboom and David Widerker have argued against the compatibilist position, if determinism is true and there is only ever one course of action available to us, we cannot be held responsible for this action.^43^ The compatibilist, they argue, has no convincing answer to the question ‘what else could I have done?’ On these grounds, the hard determinist may reject justice as fairness due to its implications for moral responsibility. The Rawlsian response as traditionally understood is not, then, entirely satisfactory.

This leads us to the second option. Can we cast hard determinists as unreasonable? This is perhaps also in line with the traditional response of political liberals. For instance, Burton Dreben states that Rawls’ aim is not to argue for a society based on the doctrine of political liberalism.^44^ Instead, it is to show that a certain ideal of constitutional liberal democracy is internally coherent. This means admitting that citizens within modern societies can be unreasonable, but that this does not undermine the internal coherence of constitutional ideals. Quong argues along similar lines.^45^ Freeman also accepts that some doctrines within many modern societies are unreasonable, offering political libertarianism as an example.^46^ That many people endorse such doctrines is not a problem that need concern us. So long as the constitutional ideals are coherent, we need not worry about those unreasonable people existing beyond the scope of these ideals. Thus, maybe hard determinists can be deemed unreasonable.

There are two reasons why I find this approach unsatisfactory. First, the aim of political liberalism is to show not only that it is internally coherent, but that it also coheres with reasonable religious doctrines. The incoherence at stake here is still at a level of abstraction; this does not only concern the sorts of beliefs people hold in modern societies. If political liberalism does not cohere with religious doctrines in the abstract, with the only way to reconcile political liberalism with religious doctrines being to deem such doctrines unreasonable, political liberalism is failing in its aim. Once we step away from this level of abstraction, however, we will be confronted by the beliefs which people do hold, which brings us to my second reason. This approach means claiming that many who endorse religious doctrines are unreasonable. The Lutheran tradition claims to have 77 million adherents across 99 countries,^47^ while in 2015, 1.8 billion people followed Islam, 24.1% of the global population.^48^ While not all of these people will necessarily hold that hard determinism is true – many Muslims may follow a reading of Islam more in line with that of Masmoudi – some followers of these religions may hold a belief in predestination that sits in tension with the Rawlsian notion of individual responsibility. Casting all of these people as unreasonable means positing that a significant proportion of people across the world are beyond the scope of political justification. On this argument, the citizen who holds the appropriate liberal values to fit within the scheme of political liberalism looks to be a rarity. On the other hand, if only a small minority of citizens were Lutherans and Muslims who endorsed hard determinism, deeming these people as unreasonable may seem like an unjust persecution of a minority.

Besides the numbers of people involved, many of these people would hold the intellectual and moral capacities to act as good citizens according to the ideal of political liberalism. Religious people who accept the truth of predestination are not necessarily hoping to impose this religious doctrine on the rest of society, nor do they reject the ideal of a fair and just society or basic rights and liberties. Where those people whose belief in predestination leads them to accept metaphysical conclusions aligned with hard determinism, there is merely a disagreement between the doctrine they endorse and the notion of moral responsibility embedded within justice as fairness. It would seem that there may be ways of including such people within the framework of political justification despite their metaphysical beliefs.

Third, a distinction could be drawn between theological determinism and metaphysical determinism. While the Lutheran might hold that salvation is dependent on divine intervention, this need not influence the Lutheran’s view on moral responsibility in this life. Thus, given the Lutheran’s belief in determinism, a compatibilist argument could be made for temporal moral responsibility in order to justify Rawls’ conception of justice to the Lutheran. However, this would commit justice as fairness to compatibilism. The Thomist, who argues that the will wills itself, or the Hindu, who similarly argues that the will creates its own karma, holds a view closer to libertarianism. The Thomist and the Hindu may object to embedding compatibilism in the principles of justice they are expected to endorse. Separating theological determinism from metaphysical determinism proves more difficult in the case of the Thomist or the Hindu, given that the theological perspective influences the way in which the temporal will is considered. This option is, therefore, not wholly adequate.

Fourth, a thinner view of justice could be proposed, one which is free of any conception of human agency, and thus coheres with libertarianism, compatibilism, and hard determinism. This holds its own problems, however, as without any perspective on human agency, a political doctrine loses sight of what is at stake. There is no longer a conception of the autonomous agent – whether this is considered as political or metaphysical autonomy – whose autonomy must be protected within the doctrine. Without this protection, the liberal may worry that credence is lent to totalitarian movements aiming to impose their doctrines on human beings as if they were automatons. Another worry may be that the loss of any perspective on moral responsibility would lead to nihilism; though principles of justice may be proposed, without moral responsibility, no one can be held accountable for failing to uphold the principles.

In sum, none of these four options appears to be satisfactory. First, resorting to the Rawlsian position that justice as fairness is neutral between reasonable metaphysical doctrines fails, as it does not account for the hard determinist being expected to accept the necessity of responsibility. Second, deeming hard determinists unreasonable means claiming that otherwise reasonable citizens should be considered as existing outside of the social contract. Third, a distinction cannot be satisfactorily drawn between metaphysical and theological determinism, as a person’s theological position may affect her perspective on the temporal will. Fourth, removing any perspective on responsibility or agency leads to further concerns regarding what is at stake in politics, and the human characteristics which ought to be protected.

## An Alternative Justification

This leaves us with the fifth option: seeking other means of justification and attempting to resolve the concerns of those who endorse opposing metaphysical doctrines. Rather than attempt to argue justice as fairness does not challenge the precepts of hard determinism, or to claim that those who endorsed hard determinism are being unreasonable, we should accept that we need to find other ways of justifying a political doctrine to those with opposing metaphysical beliefs. I argue that there is a need to recognise tacit metaphysical commitments within political doctrines, and to find ways to reconcile people with opposing metaphysical beliefs to the political doctrine. Central to this task is a recognition of the importance of fairness. With this secured, tensions between political principles and metaphysical beliefs should not matter. Establishing a social contract upon a public reason with metaphysical implications need not be an impossible task.

It might be thought that the obvious route out of this dilemma is to leave it unacknowledged. Most people obey the laws of the state in which they live regardless of their religious or philosophical beliefs. Those Lutherans and Muslims who hold a belief in predestination generally continue to obey laws premised on individual responsibility. Perhaps we should just hope that they continue to do so. However, for the contractarian, this seems unpalatable. Through public reason, the political liberal hopes that the social contract will be justified to all reasonable people. Attempting to justify a political principle while hoping that people remain ignorant of the implications of this principle seems unsatisfactory. This is particularly the case for a political doctrine seeking to attain full publicity,^49^ ensuring that all citizens understand the content of the principles of justice undergirding the constitution.

However, Smilansky may object at this point. Smilansky notes that there are good reasons for assuming that we do not have free will, at least on the libertarian conception, and that compatibilism does not necessarily resolve all the concerns we may have about free will.^50^ Nevertheless, there are also good reasons for continuing to believe in free will. If we accept hard determinism and treat people as lacking the capacity for responsibility, we will no longer be respecting them as persons. Smilansky thus views free will as an illusion worth believing in. Subjecting free will to deliberative processes may shatter this illusion. There is, then, a tension between Smilansky’s position and that of the contractarian desiring full publicity. It appears to be impossible to reconcile Smilansky’s argument for the positive role of illusion with contractarianism unless the contract itself is grounded in illusion. This does not seem a suitable solution for the Rawlsian. As Wilson, Gaines, and Hill posit,^51^ knowingly deceiving or omitting information violates Rawlsian justice, as rational people would not agree to be manipulated. Furthermore, it is unlikely parties in the original position would agree that those they represent should be manipulated to believe in something. Such an agreement would also limit citizens’ moral powers, as they would be forced to formulate their conceptions of the good and their senses of justice within this illusion. For the contractarian, citizens need to be fully informed, both in terms of the conception of justice upholding the constitution, and the ideals that form part of their own lives.

The contractarian who aims to establish full publicity cannot hope that the implications of the political principles they propose go unnoticed. Instead, these implications must be acknowledged and where disagreement arises, alternative means sought to justify the political principle. Of course, this does not mean the Rawlsian must recognise a commitment to free will within justice as fairness; the Rawlsian may well object to Smilansky’s argument. However, the Rawlsian should acknowledge that in expecting citizens to behave as morally responsible agents, the Rawlsian conception of justice is at odds with the view of those hard determinists who deny moral responsibility. If the contractarian hopes to attain full publicity, the hard determinist needs to be aware of the way in which the political principles proposed conflict with hard determinism. Other means need to be sought to justify these principles to the hard determinist.

How, then, can a political doctrine which conflicts with hard determinism be justified to the hard determinist? As Rawls states,^52^ on realising that the principles of justice promote their conception of the good, a person will become willing to endorse these principles. Perhaps, then, the metaphysical incoherence can be sidestepped, and the justification can instead appeal to a person’s self-interest. If the Lutheran can establish her church, live her life according to the principles of Lutheranism, and seek others to join her church, while her ability to do so is protected through the social contract of which she is a part, she will be willing to accept the principles undergirding this social contract. However, this is also slightly unsatisfactory. The Lutheran may argue that the importance of her metaphysical and theological views outweighs the importance of the principles of justice. Her want of eternal salvation may be of more value to her than the stability of her temporal life. She may reject justice as fairness on these metaphysical grounds. It seems at this point that the only option left is to deem such a person unreasonable. This means, however, admitting that the justice as fairness demands that a person’s metaphysical and theological beliefs are secondary to the metaphysics embedded within this conception of justice. This is the very predicament the political liberal seeks to avoid. While it seems to me that this is the most appropriate solution, further work is thus needed.

Iris Marion Young argued that Rawls underestimated the extent of disagreement in modern societies.^53^ Within this disagreement, there was little chance of reaching a consensus. These conflicts between religious perspectives on free will seem to suggest that Young was correct. This is one aspect of a religious doctrine which clashes with the metaphysical implications of a political principle. We might expect to find many others. Does this mean, then, that any social contract is an impossibility? I do not think so. If there is to be hope of reaching a consensus, however, intellectual honesty and tolerance are necessary. Rather than attempting to avoid metaphysics in the course of devising political principles, this means confronting the metaphysical implications and working towards resolving concerns that arise.

As Jean Hampton argued,^54^ the incorporation of metaphysical arguments within political deliberation is not necessarily a sign of intolerance. Providing we respect the opposing views of others, metaphysical disagreements need not cause us trouble. However, disagreement between the hard determinist who claims moral responsibility is impossible and the political liberal who states that we must be held responsible for our duties is intractable. Nevertheless, there may be ways in which consensus can be found. Through bringing the hard determinist into political deliberation and agreeing to respect the metaphysical views they propose, we increase the chances of reaching a consensus.

An alternative is to accept that the hard determinist is unreasonable for not accepting moral responsibility, but not sanctioning the hard determinist for holding this view. Jonathan Quong argues that there is no right to be unreasonable.^55^ This is because the unreasonable person uses the rights granted to them to deny the grounds on which they were granted. Central to this ground is the claim that society is based on terms of fair cooperation; those whose beliefs or activities reject such a view of society are acting unreasonably and have no right to do so.

It seems, however, that the hard determinist does not have to reject the idea of a society based on terms of fair cooperation, even if she does think moral responsibility is impossible. Nevertheless, the hard determinist might still reject a social contract premised on the truth of moral responsibility. Through this rejection, she is not necessarily rejecting the notion of a society based on terms of fair cooperation. Perhaps, then, it is this latter rejection that is problematic. A person could reject aspects of the conception of justice on which the constitution is based without wholly abandoning the idea of a fair society. Providing that the hard determinist did not challenge the idea of a fair society, she could remain within the scope of political justification. As she agrees to the importance of a fair society, she is perhaps not being unreasonable at all, despite her disagreements with the metaphysical assumptions contained in the social contract. Through keeping the hard determinist within the scope of political justification, she can present reasons for reconfiguring the idea of moral responsibility. Rather than attempt to suppress this dialectic, it can be used to arrive at a sharper understanding of the tenets of the social contract.

Thus, all that needs to be justified to the hard determinist is the importance of fairness. If this is accepted, the metaphysical disagreements need not concern us. This is, then, the most appropriate solution to the problem. A social contract is established which does not assume complete metaphysical neutrality. Instead, it is recognised that aspects of the commitment entail certain metaphysical positions. However, agreement on these metaphysical positions is not sought. It is accepted that people are free to disagree over some of the implications of the social contract. Drawing on what they believe to be metaphysically true, they are free, then, to make the case for configuring the social contract in another way. What matters is that there is a shared sense of the importance of fairness underlying political deliberation.

## Reconciling Metaphysics and Politics

This leads to a different understanding of the relationship between metaphysics and politics from that proposed by political liberals, aligning instead with Hampton’s view.^56^ Rather than attempt to avoid metaphysics in politics, imagining that the inclusion of metaphysics will exacerbate existing religious and philosophical divides, metaphysics should instead be considered openly within politics. If there are metaphysical assumptions contained within a political principle, these assumptions should be considered rather than suppressed. Before concluding, my reasons for including metaphysics within politics are elaborated.

What is ultimately integral for a social contract is a shared understanding of the importance of fairness. Fairness need not be understood in the precise way in which Rawls understands it as justice as fairness. Indeed, those such as Cohen would not agree that Rawls’ conception of fairness was fair.^57^ Thus, it is not the distributive principles which are considered fair, but the methods of deliberation. Society is thought of as based on fair terms of cooperation, and political deliberation proceeds according to rules accepted as fair. As citizens, we agree that we are all free and equal; as free and equal citizens, we owe each other equal obligations. In terms of deliberation, this means that we owe each other an equal right to be heard. Those who would deny us this right are breaking the terms of fair cooperation. This constitutes the basis of the social contract. Beginning from procedures of deliberation citizens accept as fair, conclusions are arrived at which uphold this sense of fairness. In terms of securing this consensus, the routes people take to arrive at this conclusion do not matter.

Political liberals would not necessarily disagree with this. Where I diverge is in holding that other features of the social contract do depend on the routes taken. How we think about responsibility, desert, autonomy, and even our precise interpretation of fairness will depend partially on the metaphysical views we hold to be true. For example, as Smilansky notes,^58^ Rawls’ view on desert coheres with the metaphysical views of hard determinists. Though Smilansky does not explicitly spell this out, presumably this is because hard determinists would reject distribution according to desert as people have no choice over the criteria that determines their deservingness, such as their talents, character, and acts. Libertarians and compatibilists may argue on metaphysical grounds that distribution should be devised according to desert, as they think people do play a part in formulating their own talents, characters, and acts.

Rather than attempt to establish such concepts on a metaphysically neutral basis, on this view, citizens are free to argue for a conception premised on what they believe to be metaphysically true. Those who disagree know that they can present their reasons for disagreement; if they can convince others, the concept can be reformulated. With a shared understanding of the importance of fairness at the heart of the social contract, and with citizens being guided by this sense of fairness in their deliberations, the inclusion of metaphysical perspectives need not worry us.

Thus, the Lutheran hard determinist who holds that moral responsibility is an impossibility, or the Hindu who argues the opposite are both free to draw on these beliefs within the political domain. If deliberations are guided by fair rules of procedure, and both the Lutheran and the Hindu agree to the importance of fairness, regardless of the outcome, there is no need to be concerned about the conclusion being metaphysically loaded. Whoever the conclusion disfavours, both the Lutheran and the Hindu know that they can continue to argue for their position, and they will not be disadvantaged by what they believe to be metaphysically true.

Of course, the inclusion of metaphysical reasoning within politics may lead to different understandings of fairness. As mentioned, Pereboom and Widerker argue the compatibilist cannot offer a convincing answer to the question ‘what else could I have done?’^59^ The hard determinist who argues against moral responsibility might regard it as unfair that a person is expected to form her own conception of the good or to be punished for wrongful actions, as, for the hard determinist, this person could never have done otherwise. However, this is why the inclusion of metaphysics within politics is important; the hard determinist is, then, able to present reasons for altering how we think about the way in which we formulate a conception of the good or how we answer questions of retribution for wrongdoing. Citizens, then, have more tools at their disposal in order to convince others to adopt a certain view.

However, a problem is left unresolved here: if metaphysical reasoning is permitted within political deliberation, people will be bound by the conclusions of political deliberation, regardless of whether they agree with the underlying metaphysics. Hard determinists could be expected to obey laws premised on libertarianism. Should, then, hard determinists be able to reject such laws? This would essentially give everyone the right to opt out of laws with which they disagree. Political libertarians could reject taxes which went beyond what is necessary to uphold their desired small state. Religious communities could reject secular laws. Part of the fairness underlying the social contract is agreeing to accept the conclusions reached. Rather than grant citizens the right to reject laws premised on doctrines with which they disagree, citizens should instead have the right to voice their disagreement. If hard determinists consider constitutional essentials to be premised on libertarianism, they should have the right to present reasons in support of alternatives. On convincing others of why hard determinism provides better ground on which to support constitutional essentials, amendments can be made. This changes the relationship between truth and politics within political liberalism. Instead of thinking of truth as a matter for the individual to decide, politics becomes a domain where we work towards a shared understanding of truth. While it is accepted there may be constant disagreement, it is also recognised that politics cannot be stripped entirely of its metaphysical assumptions. As we cannot make this division, we should try to settle matters on metaphysical grounds, while ensuring the mode in which we deliberate is fair. If we are asked to support a constitution with which we disagree on metaphysical grounds, it is only fair that we can present our reasons for revising the constitution on these grounds.

Whatever conclusions are reached should not, though, affect the understanding of fairness within the social contract. Whatever our stance on free will and moral responsibility, we should be able to accept that fair rules of procedure in political deliberation are necessary. It does not seem that any reasonable metaphysical perspective would alter this understanding of fairness. When we deliberate with others, we stand as equals, respect the views of opposing parties, and agree to accept the conclusion reached. This view of fairness is central to the social contract; it seems unlikely any reasonable metaphysical perspective would challenge this idea of fairness. While the contractarian may be worried that this leads to perpetual instability – allowing for metaphysical arguments within politics could lead to a stream of endless revisions to the social contract as different understandings of fairness are justified – it is the shared understanding of fairness underlying the ideal deliberative standard, seemingly immune to metaphysical challenges, that grants the social contract a degree of stability. With this in place, revisions and adjustments can be made that do not threaten stability.

This moves us closer, I argue, to a picture better representative of real-world politics. People do not, generally speaking, put their beliefs to one side when making political decisions. What they hold to be true may, to some degree, affect what they think are sensible political conclusions. The Lutheran and the Hindu may each hold political views that are influenced by what they think is metaphysically true. There is nothing necessarily wrong with this. As Andrew Murphy writes, people would not give up their beliefs due to the liberal commanding them to do so (Murphy 1998, p. 257).^60^ It also seems unlikely that either the Lutheran or the Hindu would leave their beliefs behind in the political domain merely because the political liberal demanded they did. Instead, providing there is a shared understanding of the importance of fairness underlying the social contract, metaphysical arguments can be made in support of political positions. The precise understanding of fairness may change depending on the conclusions reached, as how responsibility, desert, or autonomy are configured is likely to affect a conception of fairness. However, this need not matter if people are willing to accept the importance of fairness – broadly construed but kept within the framework of acceptable values within political liberalism – and deliberate according to procedures accepted as fair. This means being tolerant and honest in regard to opposing parties, not wishing to use the power of the state to suppress metaphysical perspectives that conflict with our own.

In sum, it appears that a social contract is attainable, even in spite of the metaphysical conflicts between religious doctrines and political principles. Being open about the metaphysical implications of the political principles we propose, we can assess where metaphysical difficulties arise. Rather than attempt to sidestep such implications, the incorporation of metaphysics within politics can allow for deeper conclusions. Through allowing for the inclusion of metaphysics within politics, we move closer to realising a consensus in which the Lutheran, Thomist, Muslim, and Hindu alike can be included, regardless of their differing metaphysical and theological beliefs.

## Conclusion

The political liberal argues that, in order to reach the necessary consensus to form a social contract, the public reasons used to support this consensus should not be metaphysical. As people hold different metaphysical perspectives, trying to base a consensus on one metaphysical perspective will lead to many refusing to endorse this perspective. However, I have argued that the reasons political liberals use to support justice as fairness do hold metaphysical implications in regard to free will. We might disagree with Smilansky’s view that justice as fairness is ‘infused with the assumption of free will’, but nevertheless, it seems clear that the hard determinist who denies moral responsibility has a view somewhat at odds with the implications of justice as fairness. There is, then, a clash between doctrines stemming from metaphysical disagreement. This could be problematic as some religious doctrines contain metaphysical perspectives aligned with hard determinism. Such religious doctrines may also disagree with the conception of moral responsibility within justice as fairness.

However, this should not undermine the formulation of a social contract. If, as the political liberal argues, there is a need for full publicity – citizens need to be aware of the content of the principles of justice undergirding the constitution – then citizens should understand the possible metaphysical implications of the principles of justice. Where tensions arise between the metaphysics entwined within the political principles proposed and a citizen’s metaphysical belief, the compromise necessary to reaching a consensus is still possible. Though the hard determinist may disagree with any political principle recognising the necessity of moral responsibility, the view of the hard determinist can still be respected, while the hard determinist may still agree to the need for a fair society. It is this agreement that secures the stability of the social contract. Providing people respect the need for a fair society, the metaphysical and theological beliefs people hold do not necessarily threaten the social contract. The hard determinist is then free to present reasons for adjusting the formulation of the social contract on the grounds of hard determinism.

Nevertheless, this is not to say that metaphysics are unimportant politically. That metaphysical beliefs may alter our political beliefs is reason to include them within politics, not to exclude them. The precise way in which we formulate political concepts may be affected by what we hold to be metaphysically true. Conclusions reached in light of such metaphysical beliefs will be lent greater depth through this support. So long as those who hold opposing beliefs are not persecuted for their beliefs, there is little problem with the incorporation of metaphysics within politics. These people can continue to hold their beliefs, and present reasons for revising conclusions reached within the political domain. The consequence of this is, then, that we need to be less worried about metaphysics within politics than is argued by political liberals.^61^

## Footnotes

1 Saul Smilansky, “Free will, Egalitarianism and Rawls”*, Philosophia 31* (1-2) (2003), pp. 127- 138.

2 John Rawls, *Political Liberalism* (New York: Colombia University Press, 2005).

3 John Rawls, “Justice as Fairness: Political not Metaphysical”, *Philosophy & Public Affairs* 14 (3) (1985), pp. 223-251.

4 Rawls, “Justice as Fairness”, p. 223.

5 Rawls, “Justice as Fairness”, p. 230.

6 Rawls, *Political Liberalism*, pp. 223-227.

7 Rawls, *Political Liberalism*, pp. 220-222.

8 Samuel Freeman, “Public Reason and Political Justifications”, *Fordham Law Review* 72 (5) (2003), pp. 2021-2072; Jonathan Quong, “The Scope of Public Reason”, *Political Studies* 52 (2) (2004), pp. 233-250.

9 John Martin Fischer, “Compatibilism”, in J.M. Fischer, R. Kane, D. Pereboom, and M. Vargas (eds.), *Four Views on Free Will* (Oxford: John Wiley & Sons, 2007).

10 Daniel Dennett, *Freedom Evolves* (London: Penguin Books, 2004).

11 Epicurus, *Letter to Menoeceus* (University of Adelaide Library, 2004); Roy Weatherford, *The Implications of Determinism* (London: Routledge, 1991).

12 Peter Van Inwagen and Allen Phillips Griffiths, *An Essay on Free Will* (Oxford: Clarendon Press, 1985).

13 Pereboom, Derk. "Defending Hard Incompatibilism", *Midwest Studies in Philosophy* 29 (2005): 228-247.

14 Robert Kane, *The Significance of Free Will* (New York: Oxford University Press, 1996).

15 Thomas Hobbes, “Of Liberty and Necessity” in V. Chappell (ed.), *Hobbes and Bramhall on Liberty and Necessity* (Cambridge: Cambridge University Press, 1999); David Hume, *An Enquiry Concerning Human Understanding*, P. Millican (ed.), (Oxford: Oxford University Press, [1748] 2008), p. 59.

16 Harry Frankfurt. "Alternate Possibilities and Moral Responsibility", *Journal of Philosophy 66* (23) (1969), pp. 829-839; Harry Frankfurt, "Freedom of the Will and the Concept of a Person", *The Journal of Philosophy 68* (1) (1971), pp. 5-20.

17 John Martin Fischer, “Compatibilism”, in J.M. Fischer, R. Kane, D. Pereboom, and M. Vargas (eds.), *Four Views on Free Will* (Oxford: John Wiley & Sons, 2007).

18 Saint Augustine, *The City of God*, trans. M. Dods (New York: Modern Library, 1957).

19 Saint Augustine, “Rebuke and Grace”, *Theological Anthropology* (1887), pp.96-108; John Michael Rist, “Augustine on Free will and Predestination”, *The Journal of Theological Studies, 20* (2) (1969), pp. 420-447.

20 Rist, “Augustine on Free will and Predestination”, p. 438.

21 Rist, “Augustine on Free will and Predestination”, p. 422; William Leonard Rowe, “Augustine on Foreknowledge and Free Will”, *The Review of Metaphysics* 18 (2) (1964), pp. 356-363.

22 Robert Kane, “The Dual Regress of Free Will and the Role of Alternative Possibilities”, *Philosophical Perspectives* 14 (2000) pp.57-79; Eleonore Stump, “Aquinas's Account of Freedom: Intellect and Will”, *The Monist* 80 (4) (1997), pp. 576-597.

23 Thomas Aquinas, *Summa Theologica, I*, Trans. Fathers of the English Dominican Province (London: Burns & Oates, 1947); Stump, “Aquinas's Account of Freedom".

24 Stump, “Aquinas’s Account of Freedom”.

25 Martin Luther, *The Bondage of the Will*, Trans. H. Cole (Peabody: Hendrickson Publishers, [1525] 2008).

26 Luther, *The Bondage of the Will*, p. 57.

27 Moshe Sokol. "Maimonides on Freedom of the Will and Moral Responsibility", *Harvard Theological Review 91* (1) (1998): 25-39.

28 William Montgomery Watt, “Free Will and Predestination in Early Islam”, *The Muslim World 36* (2) (1946), pp. 124-152.

29 Watt, “Free Will and Predestination in Early Islam”, p. 152.

30 Radwan A. Masmoudi, “What is Liberal Islam? The Silenced Majority”, *Journal of Democracy* 14 (2) (2003), pp. 40-44.

31 Kane, *The Significance of Free Will*, p. 7.

32 Kane, The Significance of Free Will, p. 7; Michael Tobias, *Life Force: The World of Jainism* (Berkeley: Asian Humanities Press, 1991).

33 Kane, *The Significance of Free Will*, p. 8.

34 Paul Hacker and Donald R. Davis, “Dharma in Hinduism”, *Journal of Indian Philosophy* 34 (5) (2006), pp. 479–496.

35 Hacker and David, “Dharma in Hinduism”, p. 490.

36 Smilansky, “Free will, Egalitarianism and Rawls”, p. 132.

37 Richard Arneson, “Rawls, Responsibility, and Distributive Justice” in M. Fleurbaey, M. Salles, J.A. Weymark, (ed.), *Justice, Political Liberalism, and Utilitarianism: Themes from Harsanyi and Rawls* (Cambridge: Cambridge University Press, 2008).

38 Gerald A. Cohen, *Rescuing Justice and Equality* (London: Harvard University Press, 2008).

39 It should be noted that not all Rawlsians reach this conclusion. Indeed, Benjamin Vilhauer is a hard determinist who uses hard determinism within the original position to ground arguments for punishment. However, Vilhauer’s commitment to hard determinism does not help resolve the problem explored in this paper. See: Benjamin Vilhauer, "Persons, Punishment, and Free Will Skepticism", *Philosophical Studies 162* (2) (2013): 143–163.

40 Jonathan Quong, *Liberalism Without Perfection* (Oxford: Oxford University Press, 2011), p. 8.

41 Samuel Freeman, *Rawls* (London: Routledge, 2007), p.295.

42 Freeman, *Rawls*, p. 295.

43 Derk Pereboom, “Hard Incompatibilism”, in J.M. Fischer, R. Kane, D. Pereboom, and M. Vargas (eds.), *Four Views on Free Will* (Oxford: John Wiley & Sons, 2007); David Widerker, “Frankfurt's Attack on the Principle of Alternative Possibilities: A Further Look”, *Philosophical Perspectives* 14 (2000), pp. 181–201.

44 Burton Dreben, “On Rawls and Political Liberalism”, in S. Freeman (ed.), The Cambridge Companion to Rawls (Cambridge: Cambridge University Press, 2003), pp. 328–329.

45 Quong, *Liberalism Without Perfection*,

46 Freeman, Samuel. "Illiberal Libertarians: Why Libertarianism is not a Liberal View." *Philosophy & Public Affairs 30* (2) (2001): 105–151.

47 Lutheran World Church, “The Lutheran World Federation: A Communion of Churches”, (2021), Available at: https://www.lutheranworld.org/content/member-churches (Accessed 11.02.2021).

48 Michael Lipka and Conrad Hackett, “Why Muslims are the World’s Fastest-Growing Religious Group”, Pew Research Centre (2017), available at: https://www.pewresearch.org/fact-tank/2017/04/06/why-muslims-are-the-worlds-fastest-growing-religious-group/ (Accessed 11.02.2021).

49 Rawls, *Political Liberalism*, pp. 66–71.

50 Smilansky, S. *Free Will and illusion*. Oxford: Oxford University Press, 2000.

51 Mark R. Wilson, Jeannie Gaines, and Ronald Paul Hill. "Neuromarketing and consumer free will*"*, *Journal of consumer affairs 42* (3) (2008): 389–410.

52 Rawls, *Political Liberalism*, pp. 144–150.

53 Iris Marion Young, “Rawls's Political Liberalism”, *Journal of Political Philosophy* 3 (2) (1995), pp. 181–190.

54 Jean Hampton, “Should Political Philosophy be done without Metaphysics?”, *Ethics* 99(4) (1989), pp. 791–814.

55 Quong, *Liberalism without Perfection*, pp. 305–312.

56 Hampton, “Should Political Philosophy be done without Metaphysics?”.

57 Cohen, *Rescuing Justice and Equality*.

58 Smilansky, “Free will, Egalitarianism and Rawls”, p. 132.

59 Pereboom, “Hard Incompatibilism”, pp. 87–92; Widerker, “Frankfurt's Attack on the Principle of Alternative Possibilities”.

60 Andrew R. Murphy, “Rawls and a Shrinking Liberty of Conscience”, *The Review of Politics* 60 (2) (1998), pp. 247–276.

61 Many thanks are owed to Carl Knight, Ben Colburn, Karen Wright, Chris Berry, Sophia Dingli, and Ding Yiheng for comments on a previous draft. I am grateful to three anonymous reviewers for their comments which allowed me to develop a more focused argument.

## References

[CR1] Arneson, R. Rawls. 2008. Responsibility, and Distributive Justice. In *Justice, Political Liberalism, and Utilitarianism: Themes from Harsanyi and Rawls*, ed. M. Fleurbaey, M. Salles, and J.A. Weymark. Cambridge: Cambridge University Press.

[CR2] Aquinas, T. *Summa Theologica, I.* Translated by Fathers of the English Dominican Province. London: Burns & Oates, 1947.

[CR3] Augustine. 1887. Rebuke and Grace. *Theological Anthropology*. pp. 96-108.

[CR4] Augustine. *The City of God*. Translated by Dods, M.. New York: Modern Library, 1957.

[CR5] Cohen, G.A. 2008. *Rescuing Justice and Equality*. London: Harvard University Press.

[CR6] Dennett, D.C. 2004. *Freedom Evolves*. London: Penguin Books.

[CR7] Dreben, B. 2003. On Rawls and Political Liberalism. In *The Cambridge Companion to Rawls*, ed. S. Freeman. Cambridge: Cambridge University Press.

[CR8] Epicurus, B. 2004. *Letter to Menoeceus*. University of Adelaide Library.

[CR9] Fischer, J.M. 2007. Compatibilism. In *Four Views on Free Will*, ed. J.M. Fischer, R. Kane, D. Pereboom, and M. Vargas, M. Oxford: John Wiley & Sons.

[CR10] Frankfurt, H.G. 1969. Alternate Possibilities and Moral Responsibility. *Journal of Philosophy* 66 (23): 829–839.

[CR11] Frankfurt, H.G. 1971. Freedom of the Will and the Concept of a Person. *The Journal of Philosophy* 68 (1): 5–20.

[CR12] Freeman, S. 2001. Illiberal Libertarians: Why Libertarianism is not a Liberal View. *Philosophy & Public Affairs* 30 (2): 105–151.

[CR13] Freeman, S. 2003. Public Reason and Political Justifications. *Fordham Law Review* 72 (5): 2021–2072.

[CR14] Freeman, S. 2007. *Rawls*. London: Routledge.

[CR15] Hacker, P., and D.R. Davis. 2006. Dharma in Hinduism. *Journal of Indian Philosophy* 34 (5): 479–496.

[CR16] Hampton, J. 1989. Should Political Philosophy be done without Metaphysics? *Ethics* 99 (4): 791–814.

[CR17] Hobbes, T. 1999. Of Liberty and Necessity. In *Hobbes and Bramhall on Liberty and Necessity*, ed. V. Chappell. Cambridge: Cambridge University Press.

[CR19] Hume, D. [1748] 2008. *An Enquiry Concerning Human Understanding*. Millican, P., ed.. Oxford: Oxford University Press.

[CR20] Kane, R. 1996. *The Significance of Free Will*. New York: Oxford University Press.

[CR21] Kane, R. 2000. The Dual Regress of Free Will and the Role of Alternative Possibilities. *Philosophical Perspectives* 14: 57–79.

[CR22] Lipka, M. and Hackett, C. 2017. *Why Muslims are the World’s Fastest-Growing Religious Group*. Pew Research Centre, 2017. https://www.pewresearch.org/fact-tank/2017/04/06/why-muslims-are-the-worlds-fastest-growing-religious-group/. Accessed 11 Feb 2021.

[CR23] Luther, M. [1525] 2008. *The Bondage of the Will*. Translated by Henry Cole. Peabody: Hendrickson Publishers.

[CR24] Lutheran World Church. *The Lutheran World Federation: A Communion of Churches*. 2021. Available at: https://www.lutheranworld.org/content/member-churches (Accessed 11.02.2021).

[CR25] Masmoudi, R.A. 2003. What is Liberal Islam? The Silenced Majority. *Journal of Democracy* 14 (2): 40–44.

[CR26] Murphy, A.R. 1998. Rawls and a Shrinking Liberty of Conscience. *The Review of Politics* 60 (2): 247–276.

[CR27] Pereboom, D. 2005. Defending hard incompatibilism. *Midwest Studies in Philosophy* 29: 228–247.

[CR28] Pereboom, D. 2007. Hard Incompatibilism. In *Four Views on Free Will*, ed. J.M. Fischer, R. Kane, D. Pereboom, and M. Vargas. Oxford: John Wiley & Sons.

[CR29] Quong, J. 2004. The Scope of Public Reason. *Political Studies* 52 (2): 233–250.

[CR30] Quong, J. 2011. *Liberalism Without Perfection*. Oxford: Oxford University Press.

[CR31] Rawls, J. 1985. Justice as Fairness: Political not Metaphysical. *Philosophy & Public Affairs* 14 (3): 223–251.

[CR32] Rawls, J. 2005. *Political Liberalism*. Expanded. New York: Columbia University Press.

[CR33] Rist, J.M. 1969. Augustine on Free will and Predestination. *The Journal of Theological Studies* 20 (2): 420–447.

[CR34] Rowe, W.L. 1964. Augustine on Foreknowledge and Free Will. *The Review of Metaphysics* 18 (2): 356–363.

[CR35] Smilansky, S. 2000. *Free Will and illusion*. Oxford: Oxford University Press.

[CR36] Smilansky, S. 2003. Free will, Egalitarianism and Rawls. *Philosophia* 31 (1-2): 127–138.

[CR37] Sokol, M. 1998. Maimonides on Freedom of the Will and Moral Responsibility. *Harvard Theological Review* 91 (1): 25–39.

[CR39] Stump, E. 1997. Aquinas’s Account of Freedom: Intellect and Will. *The Monist* 80 (4): 576–597.

[CR40] Tobias, M. 1991. *Life Force: The World of Jainism*. Berkeley: Asian Humanities Press.

[CR41] Van Inwagen, P., and A.P. Griffiths. 1985. *An Essay on Free Will*. Oxford: Clarendon Press.

[CR42] Vilhauer, B. 2013. Persons, Punishment, and Free Will Skepticism. *Philosophical Studies* 162 (2): 143–163.

[CR43] Watt, W.M. 1946. Free will and Predestination in Early Islam. *The Muslim World* 36 (2): 124–152.

[CR44] Weatherford, R. 1991. *The Implications of Determinism*. London: Routledge.

[CR45] Widerker, D. 2000. Frankfurt’s Attack on the Principle of Alternative Possibilities: A Further Look. *Philosophical Perspectives* 14: 181–201.

[CR46] Wilson, M.R., J. Gaines, and R.P. Hill. 2008. Neuromarketing and consumer free will. *Journal of Consumer Affairs* 42 (3): 389–410.

[CR47] Young, I.M. 1995. Rawls’s Political Liberalism. *Journal of Political Philosophy* 3 (2): 181–190.

